# ADAPTATION OF FACE-TO-FACE COGNITIVE ASSESSMENTS FOR TELEPHONE ADMINISTRATION: A POTENTIAL THREAT TO VALIDITY

**DOI:** 10.1093/geroni/igac059.912

**Published:** 2022-12-20

**Authors:** Jason Smith, Laura Gibbons, Dan Mungas, Maria Glymour, Laura Zahodne, Elizabeth Mayeda, Richard Jones, Alden Gross

**Affiliations:** Johns Hopkins Bloomberg School of Public Health, Baltimore, Maryland, United States; University of Washington, Seattle, Seattle, Washington, United States; University of California, Davis, Sacramento, California, United States; University of California, San Francisco, San Francisco, California, United States; University of Michigan, Ann Arbor, Michigan, United States; University of California, Los Angeles Fielding School of Public Health, Los Angeles, California, United States; Warren Alpert Medical School, Brown University, Providence, Rhode Island, United States; Johns Hopkins Bloomberg School of Public Health, Baltimore, Maryland, United States

## Abstract

Telephone-administered cognitive assessments are a cost-effective, feasible, and sometimes necessary alternative to in-person assessments. However, there is a scarcity of information in large cohort studies concerning mode effects, or differences in cognitive performance attributable to assessment method instead of underlying cognition, as a potential measurement threat. We evaluated mode effects on individual cognitive items and overall cognitive score using a population-based sample of community-living older adults aged 65-79 in the US in the 2014 Health and Retirement Study for whom interview mode was randomized (n=6825). We assessed mode differences in test means and reliability, whether mode modifies associations of cognition with criterion variables, and formal measurement invariance testing by mode. Relative to those assessed face-to-face, people assessed by telephone tended to have higher scores for memory and calculation items (0.06 to 0.013 standard deviations (SD)) and lower scores for non-memory items (-0.09 to -0.01 SD). We also found evidence that estimated cognition was significantly differentially related to IADL score depending on mode of assessment, observing a stronger association among participants completing telephone interviews. Measurement invariance testing identified the largest mode differences in memory and attention items: immediate noun recall, delayed noun recall, and serial 7s scores were higher when administered by telephone. Differences by telephone vs face-to-face mode of administration are apparent in cognitive measurement in older adults, and most pronounced for tests of memory and attention that can be easier to answer via telephone. Future investigations are warranted to further evaluate methods to correct for such differences.

